# PLGA nanoparticles engineering extracellular vesicles from human umbilical cord mesenchymal stem cells ameliorates polyethylene particles induced periprosthetic osteolysis

**DOI:** 10.1186/s12951-023-02177-7

**Published:** 2023-10-31

**Authors:** Jie Xie, Yihe Hu, Weiping Su, Sijie Chen, Jiahao Wang, Shuailong Liang, Mingyu Chen, Haoyi Wang, Tianliang Ma

**Affiliations:** 1 Department of Orthopedics, The First Affiliated Hospital, Zhejiang University School of Medicine, Hangzhou, China; 2 Department of Orthopaedics, The 3rd Xiangya Hospital, Central South University, Changsha, China; 3 Department of Ultrasound Diagnosis, Second Xiangya Hospital, Central South University, Changsha, China; 4 Department of Orthopedics, Xiangya Hospital, Central South University, 87 Xiangya Road, Changsha, 410008 Hunan China

**Keywords:** Human umbilical cord derived mesenchymal stem cells, Exosomes, PLGA, Periprosthetic osteolysis, Aseptic loosening

## Abstract

The wear particle-induced dissolution of bone around implants is a significant pathological factor in aseptic loosening, and controlling prosthetic aseptic loosening holds crucial social significance. While human umbilical cord mesenchymal stem cell-derived exosomes (HucMSCs-Exos, Exos) have been found to effectively promote osteogenesis and angiogenesis, their role in periprosthetic osteolysis remains unexplored. To enhance their in vivo application, we engineered HucMSCs-Exos-encapsulated poly lactic-co-glycolic acid (PLGA) nanoparticles (PLGA-Exos). In our study, we demonstrate that PLGA-Exos stimulate osteogenic differentiation while inhibiting the generation of reactive oxygen species (ROS) and subsequent osteoclast differentiation in vitro. In vivo imaging revealed that PLGA-Exos released exosomes slowly and maintained a therapeutic concentration. Our in vivo experiments demonstrated that PLGA-Exos effectively suppressed osteolysis induced by polyethylene particles. These findings suggest that PLGA-Exos hold potential as a therapeutic approach for the prevention and treatment of periprosthetic osteolysis. Furthermore, they provide novel insights for the clinical management of osteolysis.

**Supplementary Information:**

The online version contains supplementary material available at 10.1186/s12951-023-02177-7.

## Introduction

Total joint arthroplasty (TJA) stands as one of the most successful orthopedic procedures in the 21st century, providing a crucial approach to treating end-stage bone and joint diseases. This intervention effectively alleviates patients’ pain, restores joint function, and improves their long-term prognosis [1]. Encouragingly, long-term studies have indicated survival rates exceeding 90% after 20 years of TJA [2]. Nevertheless, aseptic loosening of implants occurs in 5–10% of cases within 15–20 years [3], primarily attributable to periprosthetic osteolysis. Periprosthetic osteolysis represents a significant cause of implant loosening post TJA, necessitating revision surgery. Osteolytic lesions associated with aseptic loosening are directly linked to the bioactive wear particles generated by the implant articulations. These wear particles infiltrate the surrounding tissue, promoting osteoclast differentiation and subsequently augmenting bone resorption [4]. Notably, polyethylene, particularly ultra-high molecular weight polyethylene (UHMWPE, PE) with a high molecular weight (2–10.5) ×10^6^ g/mol, is widely employed as a joint surface material for hip prostheses [5]. Periprosthetic osteolysis arising from PE particles within artificial joints represents a major long-term complication following TJA [6]. In fact, all hip prostheses consist of a metal femur stem, a metal or ceramic ball, and a PE acetabular cup liner, with the majority of wear debris comprising PE particles [7]. Consequently, preventing and treating PE-induced periprosthetic osteolysis hold paramount clinical significance.

In comparison to bone marrow-derived mesenchymal stem cells (BMSCs), human umbilical cord mesenchymal stem cells (HucMSCs) offer a non-invasive separation method and avoid ethical concerns. HucMSCs possess lower immunogenicity, faster self-renewal ability, more stable doubling time, and higher proliferation capacity, making them highly promising for tissue damage repair, regeneration, and stem cell therapy [8]. However, the clinical application of HucMSCs has certain limitations, necessitating the exploration of cell-free methods that provide comparable yield and efficacy. Exosomes, endogenous extracellular vesicles with a diameter of 40-160 nm, contain proteins, lipids, and nucleic acids. They are derived from cells or tissues and exhibit excellent safety, low immunogenicity, and remarkable membrane-binding properties. These characteristics contribute to their favorable biocompatibility, stability, and ability to traverse the blood-brain barrier [9]. As cell-derived products, exosome-based treatments do not trigger rejection, harmful differentiation, or malignant transformation [10]. Studies have demonstrated that HucMSCs-derived exosomes (HucMSCs-Exos, Exos) retain the advantages of HucMSCs and are more amenable to clinical application [11]. Currently, HucMSCs-Exos finds widespread use in bone repair and reconstruction. For instance, Yang et al. revealed that HucMSCs-Exos, incorporated within a compound hydrogel, significantly enhanced osteogenesis and angiogenesis while expediting bone defect repair [12]. Similarly, Xue et al. demonstrated that HucMSCs-Exos facilitated kidney injury reduction and accelerated wound healing in diabetic patients by promoting angiogenesis [13–15]. Furthermore, HucMSCs-Exos exhibit immunomodulatory effects. Ti et al. discovered that pre-treated HucMSCs-Exos enhanced macrophage M2 polarization and alleviated inflammation [16]. However, the role of HucMSCs-Exos in periprosthetic osteolysis remains unexplored.

To address and mitigate aseptic loosening of prostheses, two fundamental scientific challenges must be tackled: (1) Effective Drug Selection: The treatment should involve a drug that not only effectively inhibits the bone lysis reaction but is also safe and devoid of ethical concerns. (2) Sustained Drug Delivery: The therapy should enable the establishment of a sustained and efficacious drug concentration in the vicinity of the prosthesis. In the current research, poly lactic-co-glycolic acid (PLGA) stands out as a commonly employed drug carrier in nanoparticle-based studies. This choice is attributed to its excellent biocompatibility, ease of production, and efficient sustained release properties [17, 18]. For instance, Li et al. utilized PLGA/pDA scaffolds for the gradual and continuous release of human adipose stem cells-derived exosomes, and the exosome sustained release system significantly enhanced bone defect repair [19]. Similarly, Zhao et al. demonstrated that magnetic PLGA microspheres loaded with superparamagnetic iron oxide nanoparticles (SPIONs) contributed to bone defect reconstruction through the regulation of BMSCs under the influence of an external magnetic field [20]. Building upon the promising precedents of PLGA drug-loaded nanoparticles preparation, our research group successfully fabricated HucMSCs-Exos-encapsulated PLGA nanoparticles (PLGA-Exos) using an emulsification solvent volatilization method. This gentle preparation process ensured the preservation of exosome integrity and prevented denaturation during the encapsulation process. The outcome was the successful creation of PLGA-Exos, forming a stable sustained release system. We conducted comprehensive in vivo and in vitro experimental studies on PLGA-Exos to ascertain their efficacy in preventing and treating aseptic loosening of prostheses. Our findings unequivocally indicate that the PLGA-Exos sustained release system can gradually release exosomes and maintain a high local exosome concentration in the context of osteolysis. This concentration promotes the osteogenic differentiation of BMSCs while concurrently inhibiting the osteoclastic differentiation of macrophages. Moreover, the results of in vivo experiments demonstrated that the PLGA-Exos group outperformed the Exos group alone in inhibiting PE particles-induced calvarial osteolysis. This study constitutes a pioneering endeavor that introduces novel methods and concepts for averting and managing aseptic loosening of prostheses following joint replacement. Its profound theoretical significance and the potential application value it offers underscore its importance in advancing the field of orthopedic research and clinical practice.

## Materials and methods

### Regents and materials

ExoQuick-TC (System Biosciences, EXOTC50A-1, California, USA), BCA protein assay kit (New Cell & Molecular Biotech Co., Ltd, WB6501, Suzhou, China), PLGA (Jinan Daigang Biomaterial Co., Ltd., Jinan, China), 5×SDS protein loading buffer (Beyotime, P0015L, Shanghai, China), PVDF membrane (Millipore, ISEQ00010/IPVH00010, USA), DII (Beyotime, C1036, Shanghai, China), DIO (Beyotime, C1038, Shanghai, China), DAPI (Biosharp, 28718-90-3, Anhui, China), Cell Counting Kit-8(MedChemExpress, HY-K0301, USA), Calcein-AM/PI Double Stain Kit (Yeasen, 40747ES76, Shanghai, China), BCIP/NBT Alkaline Phosphatase Color Development Kit (Beyotime, C3206, Shanghai,China), RANKL (R&D, 462-TEC-010/CF, Minnesota, USA), DIR dye-labeled (Yeasen, 40726ES10, Shanghai, China), anti-CD9 (Abcam, ab236630, UK), anti-CD63(Abcam, ab134045, UK), anti-TSG101 (Abcam, ab125011, UK), anti-Calnexin (Abcam, ab22595, UK), anti-OCN(Abcam, ab93876, UK), anti-IL-6 (Abcam, ab290735, UK), and anti-TNF-α (Abcam, ab1793, UK).

### 2.2 Cell culture

This study received approval from the Ethics Review Committee of Xiangya Hospital, Central South University (approval number: 2019030526), and informed consent was obtained from all subjects prior to sample collection. HucMSCs were isolated from fresh umbilical cord samples as previously described [21] and maintained in a stem cell culture medium (Cyagen, Guangzhou, China). BMSCs were extracted from the femur and tibial bone marrow of male C57BL/6J mice aged 4–6 weeks and maintained with alpha modification (α-MEM) and fetal bovine serum (FBS, Gibco, New York, USA) and penicillin-streptomycin solution (Procell, Wuhan, China) and RAW264.7 culture medium includes DMEM high glucosebase medium (Gibco, New York, USA), FBS (Procell, Wuhan, China), and penicillin-streptomycin solution.

### Exosome isolation

The extraction of exosomes was performed using a previously reported method [22]. When HucMSCs reached 70–80% confluence, the cells were washed three times with PBS (Biosharp, Anhui, China) and cultured in a complete medium without exosomes for an additional 48 h. The collected cell supernatant was subjected to centrifugation and filtered through a 0.22 μm filter (Millipore, USA). The filtered supernatant was then added to an ultrafiltration tube (Millipore, USA) and centrifuged (4000 g/20min) until the volume was reduced to 1mL. Following the completion of the ultrafiltration, the supernatant was washed three times with PBS. ExoQuick-TC was added at a ratio of 1:5, mixed thoroughly by inverting, and incubated overnight at 4 °C. Subsequently, the mixture was centrifuged at 1500 g for 30 min, the supernatant was discarded, and an appropriate amount of PBS was added to resuspend the pellet based on the precipitation amount. The obtained exosome sample was quantified using the BCA protein assay kit. The particle size and zeta potential of the exosomes were determined using dynamic light scattering (DLS) (Malvern, UK), and the ultrastructure of the exosomes was observed using transmission electron microscopy.

### Preparation of PLGA and PLGA-Exos

PLGA nanoparticles were prepared using the single emulsification method [23, 24]. Firstly, PLGA was dissolved in dichloromethane, and then a 4% cold poly (vinyl alcohol) (PVA) solution was added to the PLGA solution. The mixture was subjected to ultrasonic shock for 4 min (150 W, 0-5 s-0s) using an ultrasonic homogenizer (Sonics, VCX150, USA) to facilitate emulsification. Subsequently, the organic solvent was volatilized by stirring on a magnetic mixer for 2–4 h (Extend stirring time as necessary to ensure complete evaporation of organic solvent). After volatilization, the liquid was centrifuged at 10,000 rpm for 10 min, and the resulting precipitate was washed three times by ddH_2_O.

For the preparation of PLGA-Exos, PLGA, Exos were dissolved completely in dichloromethane. Then, pre-cooled 4% w/v PVA solution was added. The mixture was sonicated for 4 min (150 W, 0-5 s-0s) using an ultrasonic homogenizer and stirred for 2–4 h to allow dichloromethane evaporation (Extend stirring time as necessary to ensure complete evaporation of organic solvent). The resulting nanoparticles were washed three times with deionized water and kept for further use. The particle size and zeta potential of the nanoparticles were determined using DLS (Malvern, UK), and the ultrastructure was observed using transmission electron microscopy. The in vitro sustained-release ability of PLGA-Exos was quantitatively assessed using the BCA protein assay kit. The prepared PLGA-Exos nanoparticles and PLGA/Exos mixture were suspended in 500 µL of PBS and gently shaken in a 37℃ shaker. After centrifugation every 24 h at 10,000 rpm for 10 min, 100 µL of supernatant was collected for BCA protein quantification. Subsequently, 100 µL of PBS was added, and the suspension was slowly shaken at 37℃. After two weeks, the sustained release efficiency of the PLGA-Exos and PLGA/Exos mixture was calculated. Exos loading was calculated according to the following equation [25].

Loading efficiency (%) = Weight of Exos in the nanoparticles/ Weight of nanoparticles ×100%.

### Western blot analysis

To extract the total proteins from PLGA, Exos, and PLGA-Exos, a lysate PMSF: RIPA (1:100) solution was prepared. BCA protein quantitative analysis was performed to determine the total protein concentration. Subsequently, 5×SDS protein loading buffer was added to the samples to ensure complete cell lysis. The mixture was thoroughly mixed and denatured at 95 °C for 10 min. A 10% SDS polyacrylamide gel was prepared, and 20 µg of protein was loaded for electrophoresis (100 V, 90 min). After electrophoresis, the proteins on the gel were transferred to a PVDF membrane (400mA, 20 min) and subsequently blocked using 5% bovine serum albumin (BSA, BioFroxx, Germany). The PVDF membrane was then incubated overnight at 4 °C with anti-CD9, anti-CD63, anti-TSG101, and anti-Calnexin antibodies. Following this, the PVDF membrane was incubated with an HRP-binding antibody at room temperature for 90 min. Finally, the signal was detected using the ChemiDoc XRS (Bio-Rad, USA).

### Laser confocal microscopy analysis

To further confirm the presence of PLGA-Exos, confocal laser scanning microscopy (CLSM) was employed. Prior to the preparation of PLGA-Exos, PLGA was stained with DII (Beyotime, Shanghai, China) while Exos were stained with DIO (Beyotime, Shanghai, China). RAW264.7 cells were cultured overnight in advance and then exposed to the stained PLGA-Exos for 6 h. Following this, the culture medium was aspirated and the cells were washed three times with PBS at room temperature (90 rpm, 5 min). The cells were fixed with 4% paraformaldehyde (Servicebio, Wuhan, China) at 37 °C for 10 min, and DAPI staining was performed for 10 min at room temperature. CLSM was utilized for the subsequent observation and photography of the samples.

### CCK8 and the live/dead staining

Following the method previously reported [26], BMSCs were extracted from the femur and tibial bone marrow of male C57BL/6J mice aged 4–6 weeks. RAW264.7 cells were purchased from Procell in Wuhan. For the experiments, 5 × 10^3^ BMSCs and RAW264.7 cells were seeded in 96-well plates and treated with various concentrations of Exos and PLGA-Exos for 48 h after cell adhesion. The cytotoxicity of Exos and PLGA-Exos was assessed using the cell counting kit-8 and calcein-AM/PI double stain kit.

### Osteogenic differentiation

To assess the impact of Exos and PLGA-Exos on the osteogenic differentiation of BMSCs, 1 × 10^4^ BMSCs were seeded in each well of 48-well plates. The osteogenic induction medium comprised 90% α-MEM (Invitrogen, California, USA), 10% FBS (Gibco, New York, USA), 10 nmol/L dexamethasone (Sigma-Aldrich, USA), 10 mmol/L β-glycerol phosphate (Sigma-Aldrich, USA), and 100 µmol/L Vitamin C (Invitrogen, California, USA). After 3 days of culture with 100 µg/mL Exos and PLGA-Exos, a BCIP/NBT alkaline phosphatase color development kit was performed, and the supernatant was collected for measuring Ca^2+^ levels and ALP activity. Subsequently, after 7 days of osteogenic induction, ALP staining was repeated. Alizarin Red S (ARS) staining and Sirius red staining were conducted 14 days after induction, and absorbance was measured following elution with the appropriate eluent.

### Reactive oxygen species

In each well of the 48-well plate, 5 × 10^4^ RAW264.7 cells were seeded. Once the cells had firmly adhered to the plate, they were cultured in an osteoclast induction medium containing 1 mg/mL PE and 50 ng/mL RANKL (P + R) (R&D Systems, Minnesota, USA). Cells were treated with 300 µg/mL Exos and PLGA-Exos for 12 h. To assess the impact of Exos on ROS production, a reactive oxygen reactive dye, DCFH-DA(Boxbio Science & Technology Co., Ltd, Beijing, China), was employed.

### Osteoclast differentiation

In each well of the 48-well plate, 1 × 10^4^ RAW264.7 cells were seeded. Once the cells had firmly adhered to the plate, they were cultured in an osteoclast induction medium containing 1 mg/mL PE and 50 ng/mL RANKL (P + R). The intervention was performed using 300 µg/mL Exos and PLGA-Exos. After 3 days of osteoclast induction, the levels of IL-6 and TNF-α inflammatory factors were assessed by collecting cell supernatant from each group. On day 5 of osteoclast induction, the number and morphology of osteoclasts were evaluated using the tartrate-resistant acid phosphatase (TRAP) staining kit (Sigma-Aldrich, USA), and the stained purplish-red osteoclasts were observed under an inverted microscope (Leica, Germany).

### Mouse calvarial osteolysis

This experimental procedure was approved by the Experimental Animal Management Committee of Central South University and conducted in accordance with the principles outlined in the “Guidelines for Ethical Review of Experimental Animal Welfare.“ Following a previously reported protocol [27], male C57BL/6J mice aged 8–10 weeks were utilized to establish the PE particles-induced calvarial osteolysis model. All surgical instruments and PE particles were thoroughly sterilized, the mice were properly anesthetized and monitored for anesthesia effectiveness, and a one-centimeter incision was made along the midsagittal line. After the surgical blade scraped off the calvarial periosteum, sterilized PE particles were evenly applied to the skull surface (in the sham operation group, only the periosteum was removed without covering PE particles). Based on the experimental requirements, the mice were divided into the following groups: sham group, PE group, PE + PLGA group, PE + Exos group, and PE + PLGA-Exos group. On the second day of the procedure, PBS, PLGA, Exos (200 µg Exos, 100 µL), and PLGA-Exos (200 µg Exos, 100 µL) were locally injected, followed by weekly injections. After one month of modeling, the mice were euthanized. The skull pieces and major organs (heart, liver, spleen, lung, kidney, and brain) were extracted and fixed with paraformaldehyde for two days.

### In vivo imaging

A calvarial osteolysis model induced by PE particles was established in male C57BL/6J mice aged 8–10 weeks. The mice were subjected to topical injections of DIR dye-labeled Exos (200 µg Exos, 100 µL) and PLGA-Exos (200 µg Exos, 100 µL) one day after the modeling procedure, while an equal amount of PBS solution was injected as a control. Fluorescence images were captured at 1, 2, 3, 4, 5, and 7 days using an in vivo imaging system (IVIS, Caliper, USA). After 7 days, the mice were sacrificed to analyze the distribution of fluorescence in the skull and organs, and the IVIS system was utilized to measure the fluorescence intensity.

### Micro-CT analysis and bone histomorphometry

The fixed mouse skull slices were subjected to evaluation using micro-computed tomography (micro-CT) (SkyScan 1176; SkyScan, Aartselaar, Belgium). After scanning, 3D reconstruction was performed, and a region of interest (ROI) was selected for the measurement of bone surface/bone volume (BS/BV), bone volume/total volume (BV/TV), and total porosity. Following decalcification in 10% EDTA (Servicebio, China) for one week, the skull slices were embedded in paraffin for histological examination. Thin sections of 5 μm were stained with leukocyte acid phosphatase TRAP (Sigma-Aldrich, USA) and hematoxylin-eosin (H&E, Wako), and then observed and photographed under a microscope (Leica, Germany).

### Immunohistochemistry staining

The sections were placed in a slide rack and baked in a 60℃ incubator for 60–120 min, followed by dewaxing in three stages. Antigen retrieval was performed to rehydrate the sections, and endogenous peroxidase activity was quenched through washing. The primary antibodies, including anti-OCN (Abcam, UK), anti-IL-6 (Abcam, UK), and anti-TNF-α (Abcam, UK), were diluted in 1% BSA and incubated with the sections overnight at 4℃. After warming to 37℃ for 45 min and subsequent washing, the sections were incubated with a secondary antibody (1:200; Abcam, UK) in a 37℃ humid chamber for 30 min. Images were captured using a light microscope (Nikon, Japan). The staining intensity and the number of positive cells were quantified using Image Pro Plus 6.0 software.

### Statistical analysis

The data in our study were replicated three times. GraphPad Prism 8 and ImageJ software were employed for data analysis. Statistical differences between the two groups were determined by a two-tailed Student’s t-test. Multiple groups were compared using a one-way analysis of variance (ANOVA). A P value less than 0.05 was deemed statistically significant, whereas “ns” indicated no significant difference. The significance levels were represented as follows: *P < 0.05, **P < 0.01, and ***P < 0.001.

## Results

### Extraction and characterization of human umbilical cord mesenchymal stem cells-derived exosomes

**Fig. 1:**
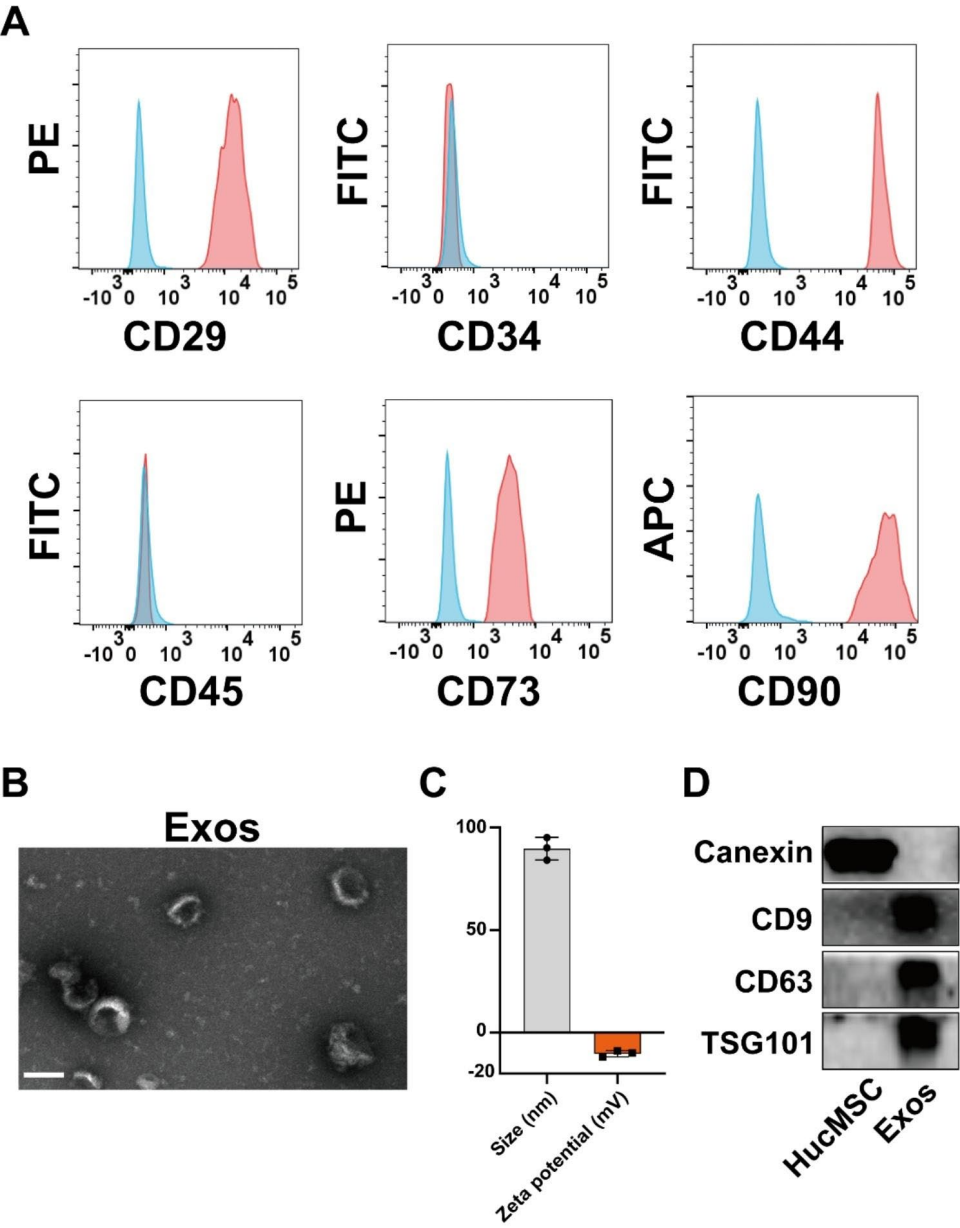
**Extraction and characterization of exosomes from human umbilical cord mesenchymal stem cells.** (**A**) Flow cytometry analysis of the typical surface markers in HucMSCs. Blue curves: the isotype controls; Red curves: the test samples. (**B**) TEM images of Exos. Scale bar, 100 nm. (**C**) Size distribution (PDI = 0.14) and zeta potential of Exos were measured by dynamic light scattering (n = 3). (**D**) Typical surface markers expressed on HucMSCs and Exos were determined by Western blot analysis (Calnexin: 90 kDa, CD9: 25 kDa, CD63: 26 kDa, TSG101: 45 kDa)

HucMSCs were obtained from human umbilical cords. Flow cytometry analysis was employed to confirm the stem cell characteristics of HucMSCs, which aligned with previous reports [21] (Fig. 1A). Specifically, HucMSCs exhibited positive expression for CD29, CD44, CD73, and CD90, while they were negative for CD34 and CD45. HucMSCs-Exos were isolated from the collected supernatant and subjected to characterization and identification using transmission electron microscopy, DLS for particle size analysis, and western blot analysis. Transmission electron microscopy revealed that these nanoscale vesicles exhibited a concave disc-shaped, cup-shaped, or disc-shaped morphology, with an average particle size of approximately 90 nm (Fig. 1B). Furthermore, DLS analysis determined the average particle size of the exosomes to be around 95 nm, with a zeta potential of approximately − 15mV (Fig. 1C). Western Blot analysis confirmed the presence of exosome-specific marker proteins, including CD9, CD63, and TSG101, while the intracellular protein calnexin was not detected (Fig. 1D). These findings collectively demonstrate the successful isolation of HucMSCs-Exos.

### Construction and characterization of PLGA-Exos

**Fig. 2:**
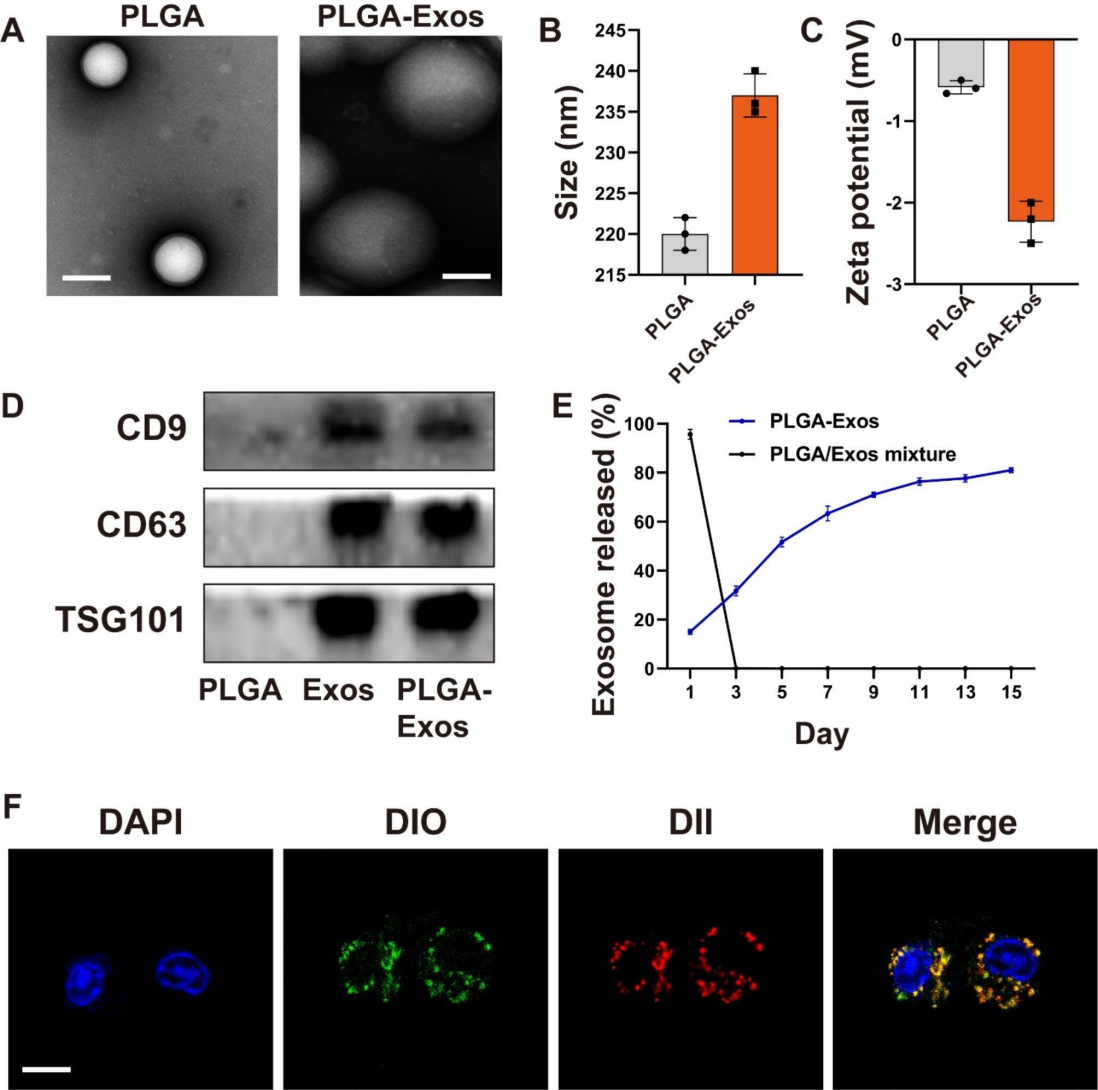
**Construction and characterization of PLGA-Exos.** (**A**) TEM images of PLGA and PLGA-Exos. Scale bar, 200 nm. (**B**) Size distribution (PDI = 0.15, 0.18) and (**C**) zeta potential of PLGA and PLGA-Exos were measured by dynamic light scattering (n = 3). (**D**) Typical surface markers expressed on PLGA, Exos, and PLGA-Exos were determined by Western blot analysis (CD9: 25 kDa, CD63: 26 kDa, TSG101: 45 kDa). (**E**) Quantitative analyses of the releases of exosomes from PLGA-Exos and PLGA/Exos mixture over time. (**F**) Typical confocal images of RAW264.7 incubated with M2-BMSCs-Exos, (DiO-labeled Exos (green) and DII-labeled PLGA (red)). Scale bar, 10 μm

In order to enhance the in vivo applicability of Exos, we prepared the Exos-encapsulated PLGA nanoparticles (PLGA-Exos). Transmission electron microscopy analysis revealed that PLGA nanoparticles exhibited homogenous spheroidal morphology, and the PLGA-Exos were dispersed within the PLGA structure (Fig. 2A). The particle size of PLGA-Exos slightly increased compared to PLGA alone, while the zeta potential decreased (Fig. 2B, C). Western Blot analysis confirmed the expression of exosome-specific proteins CD9, CD63, and TSG101 in PLGA-Exos (Fig. 2D) and coomassie brilliant blue staining results showed that PLGA Exos retained the protein components of Exos (Figure.S1). To assess the sustained-release capability of PLGA-Exos, we employed the BCA protein quantification method of PLGA-Exos and PLGA/Exos mixture. The results indicated that PLGA-Exos achieved a sustained-release capacity of over 80% for approximately 14 days, demonstrating its favorable sustained-release performance (Fig. 2E), and the loading efficiency of Exos was 8.89%. Furthermore, we validated the prepared PLGA-Exos using CLSM, where the red PLGA signal overlapped with the green Exos signal around the nucleus (Fig. 2F). These findings confirmed the successful preparation of PLGA-Exos.

### PLGA-Exos enhanced the osteogenic differentiation and inhibited osteoclast formation in vitro

**Fig. 3:**
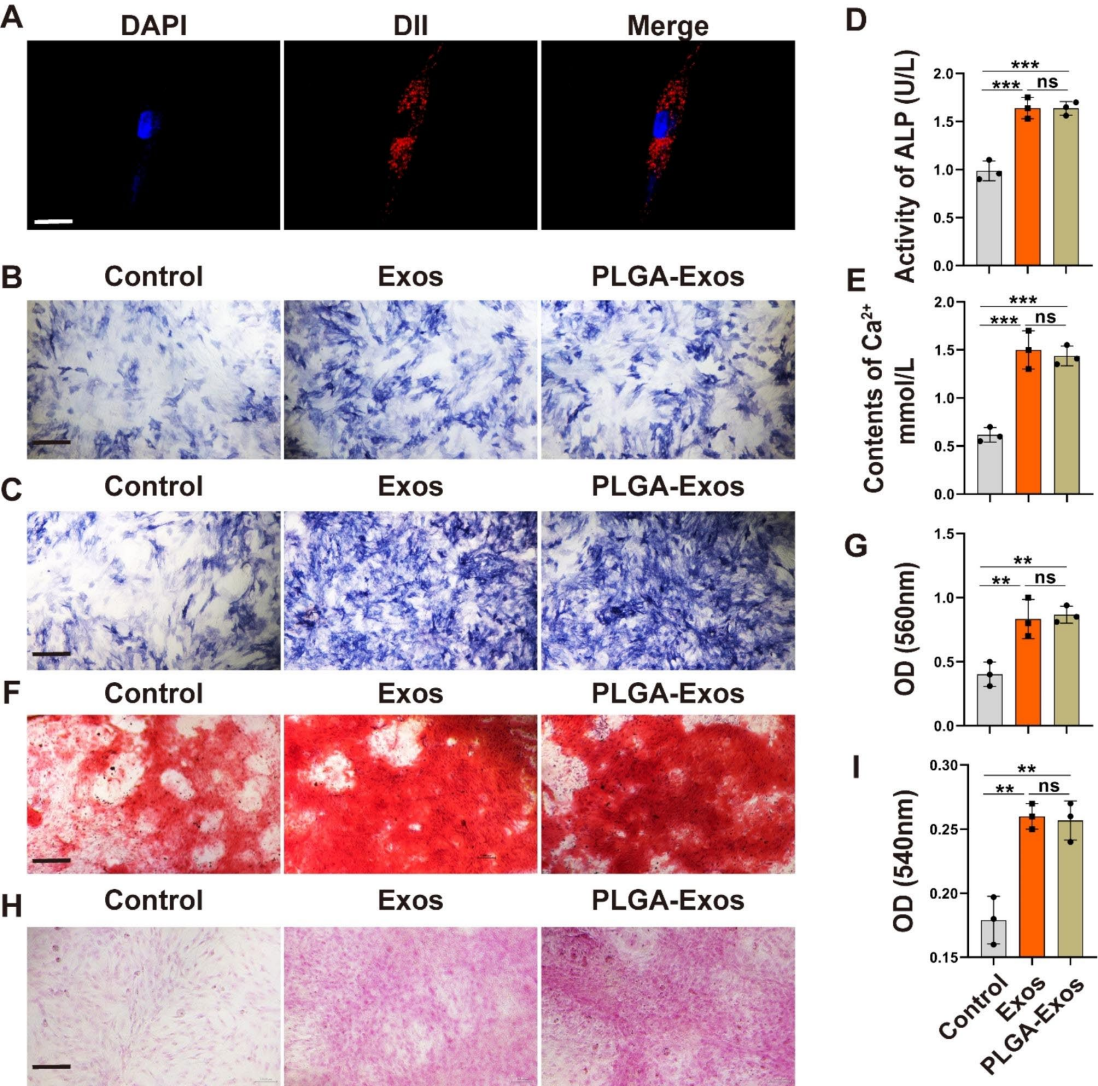
**PLGA-Exos enhanced the osteogenic differentiation in vitro.** (**A**) Representative immunofluorescence images showing the internalization of DII-labeled Exos (red) by BMSCs and the cell nuclei of BMSCs were stained with DAPI (blue). Scale bar: 20 μm. (**B**) ALP activity assays of BMSCs after 3 days and (**C**) 7 days of osteogenic induction. Scale bar, and 400 μm. (**D**, **E**) Quantitative analyses of ALP and Ca^2 +^ levels in medium from BMSCs receiving Exos treatments. (**F**-**I**) ARS staining and Sirius red staining of BMSCs after 14 days of osteogenic induction and determination of the absorbance of the eluate after ARS staining and Sirius red staining. Scale bar, 200 μm (F) and 400 μm (H). (n = 3 per group, ANOVA, *P < 0.05, ** *P* < 0.01, *** *P* < 0.001)

We initiated our investigation by examining the impact of **PLGA-Exos** on the osteogenic differentiation of BMSCs obtained from the femoral and tibial bone marrow of male C57BL/6J mice aged 4–6 weeks. BMSCs exhibited for negative for CD45 and positive for CD29, CD44, and CD90 (Figure.S2). To assess the uptake capacity of BMSCs, we labeled Exos with the lipophilic dye DII (red fluorescence) and stained the BMSC nuclei with DAPI. Through CLSM, a substantial presence of red Exos nanoparticles surrounding the BMSCs nuclei was observed, indicating the efficient uptake ability of Exos by BMSCs (Fig. 3A). To further investigate the osteogenic-promoting potential of PLGA-Exos, BMSCs were treated with an osteogenic induction medium supplemented with Exos and PLGA-Exos. We conducted ALP staining on days 3 and 7, and the results revealed a significant increase in ALP activity in BMSCs stimulated by Exos and PLGA-Exos (Fig. 3B, C). Additionally, on day 3 of osteogenic induction, we measured the levels of ALP activity and Ca^2+^ in the supernatant, and the findings demonstrated a significant elevation in both parameters following Exos and PLGA-Exos intervention (Fig. 3D, E). After 14 days of ARS and Sirius red staining (Fig. 3F-I), enhanced extracellular matrix mineralization and collagen production were observed in the Exos and PLGA-Exos group compared to the control group, signifying the osteogenic differentiation-promoting capability of Exos and PLGA-Exos in BMSCs.

**Fig. 4:**
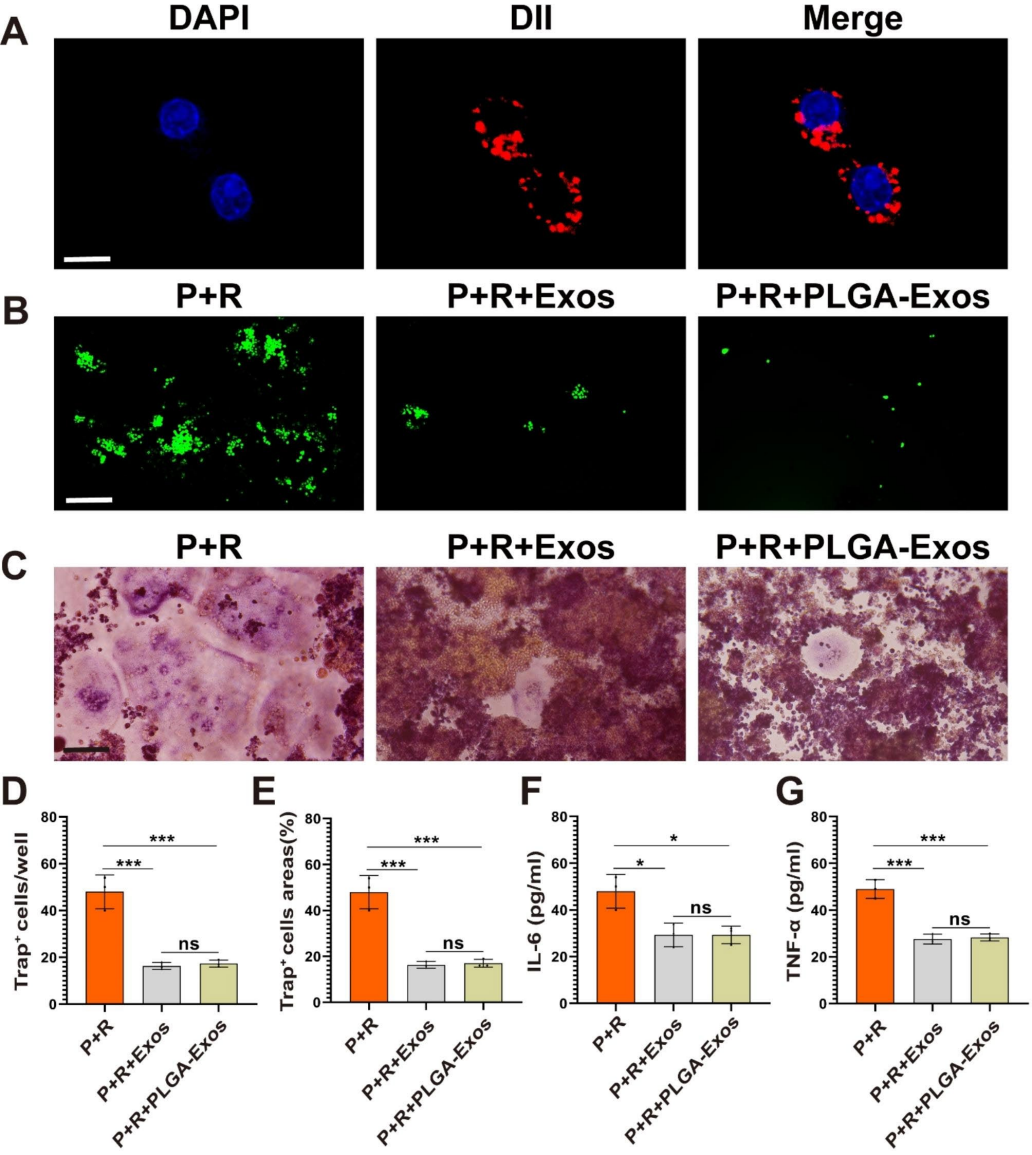
**PLGA-Exos inhibited Osteoclast Formation in vitro.** (**A**) Representative immunofluorescence images showing the internalization of DII-labeled Exos (red) by RAW264.7 cells and the cell nuclei of RAW264.7 cells were stained with DAPI (blue). Scale bar: 10 μm. (**B**) The production of ROS (green) was assayed using a fluorescence microscope. Scale bar: 200 μm. (**C**-**E**) The number of TRAP^+^ multinucleated (> 3 nuclei) osteoclasts and the TRAP^+^ cell areas were determined. (**F**, **G**) The IL-6 and TNF-α levels in the medium (RAW264.7 cells) were determined with ELISA. (n = 3 samples per group, ANOVA, *P < 0.05, ** *P* < 0.01, *** *P* < 0.001)

Furthermore, we examined the effect of PLGA-Exos on RAW264.7 cells, specifically their differentiation into osteoclasts. Through CLSM, we observed red nanoscale particles (Exos) around the nuclei (blue signal) of RAW264.7 cells, indicating the efficient uptake ability of Exos by RAW264.7 cells (Fig. 4A). Intracellular levels of ROS play a crucial role in osteoclast differentiation. ROS serve as secondary signals in osteoclasts and are produced during RANKL-induced osteoclastgenesis [28]. Thus, we initially investigated the effect of PLGA-Exos on ROS production (Fig. 4B). By employing the ROS-sensitive dye DCFH-DA and fluorescence microscopy, we observed that Exos and PLGA-Exos reduced ROS levels in RAW264.7 cells following PE + RANKL (P + R) intervention. After 7 days of P + R intervention, TRAP staining was utilized to directly assess the impact of PLGA-Exos on osteoclast differentiation in RAW264.7 cells (Fig. 4C-E). The results indicated that Exos and PLGA-Exos intervention significantly reduced the number and area of osteoclasts. The supernatant was collected for the evaluation of inflammatory factors, and the ELISA results demonstrated that Exos and PLGA-Exos reduced the production of inflammatory factors (IL-6 and TNF-α) following P + R intervention (Fig. 4F, G). In conclusion, our findings indicate that PLGA-Exos can inhibit osteoclast differentiation in RAW264.7 cells induced by P + R.

We evaluated the cytotoxicity of Exos using CCK-8 detection and live/dead cell double staining. Exos and PLGA-Exos (0 µg/mL, 100 µg/mL, and 300 µg/mL) promote the proliferation of BMSCs and RAW264.7 to a certain extent without significant cytotoxicity (Figure.S3, S4).

### Biodistribution of PLGA-Exos in mice with PE particles-induced mouse calvarial osteolysis

**Fig. 5:**
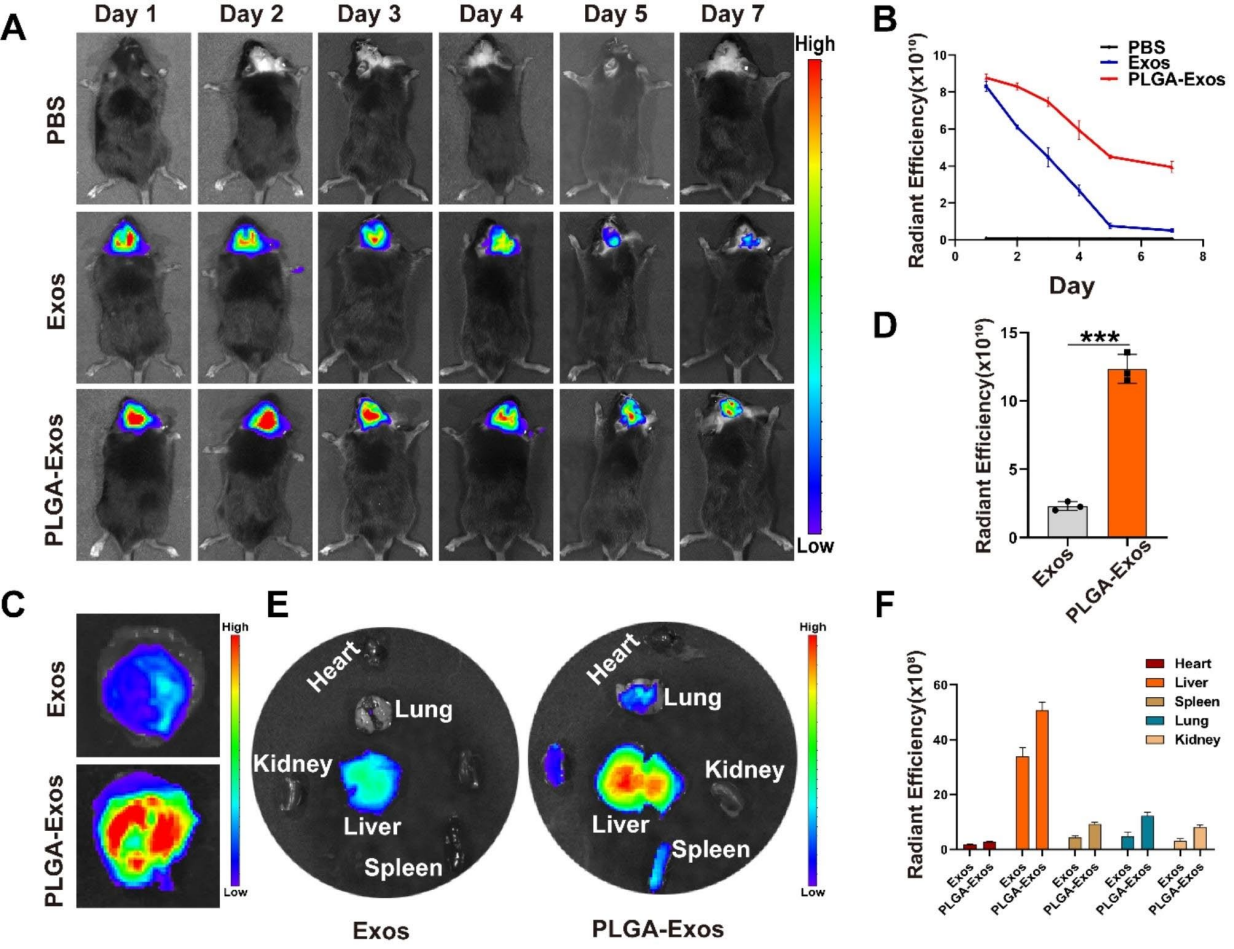
**Biodistribution of PLGA-Exos in mice with PE particles-induced mouse calvarial osteolysis.** (**A**, **B**) Representative fluorescence images after local injections of DiR-labeled Exos (200 µg) and PLGA-Exos (200 µg), and quantification of the fluorescent intensity. (**C**, **D**) Representative IVIS images showed the distribution of the calvarial region of the mouse and the quantification of the fluorescent intensity. (n = 3 samples per group, t-test, *P < 0.05, ** *P* < 0.01, *** *P* < 0.001) (**E**, **F**) Representative IVIS images showed the distribution of Exos and PLGA-Exos in the main organs after PE particle-induced calvarial osteolysis. (n = 3 samples per group, *P < 0.05, ** *P* < 0.01, *** *P* < 0.001)

To evaluate the effectiveness of PLGA-Exos in vivo, we established a mouse model of PE particles-induced mouse calvarial osteolysis. On the second day of modeling, locally injected DIR-labeled Exos and PLGA-Exos were examined for fluorescence signals using an IVIS. The results revealed higher fluorescence signals on the skull surface of the PLGA-Exos group compared to the Exos group on day 1, day 2, day 3, day 4, day 5, and day 7 post-injection (Fig. 5A). PLGA-Exos gradually released Exos, maintaining a sustained high fluorescence signal by day 7 (Fig. 5B). After seven days, the mice were euthanized, and the skull slices and major organs were collected. The fluorescence signals of the isolated skull slices and major organs were recorded and analyzed (Fig. 5C-F), demonstrating higher fluorescence signals in the PLGA-Exos group compared to the Exos group. These outcomes substantiated the successful preparation of PLGA-Exos and confirmed the sustained release of Exos in vivo.

### PLGA-Exos alleviates PE particles-induced mouse calvarial osteolysis in vivo

**Fig. 6:**
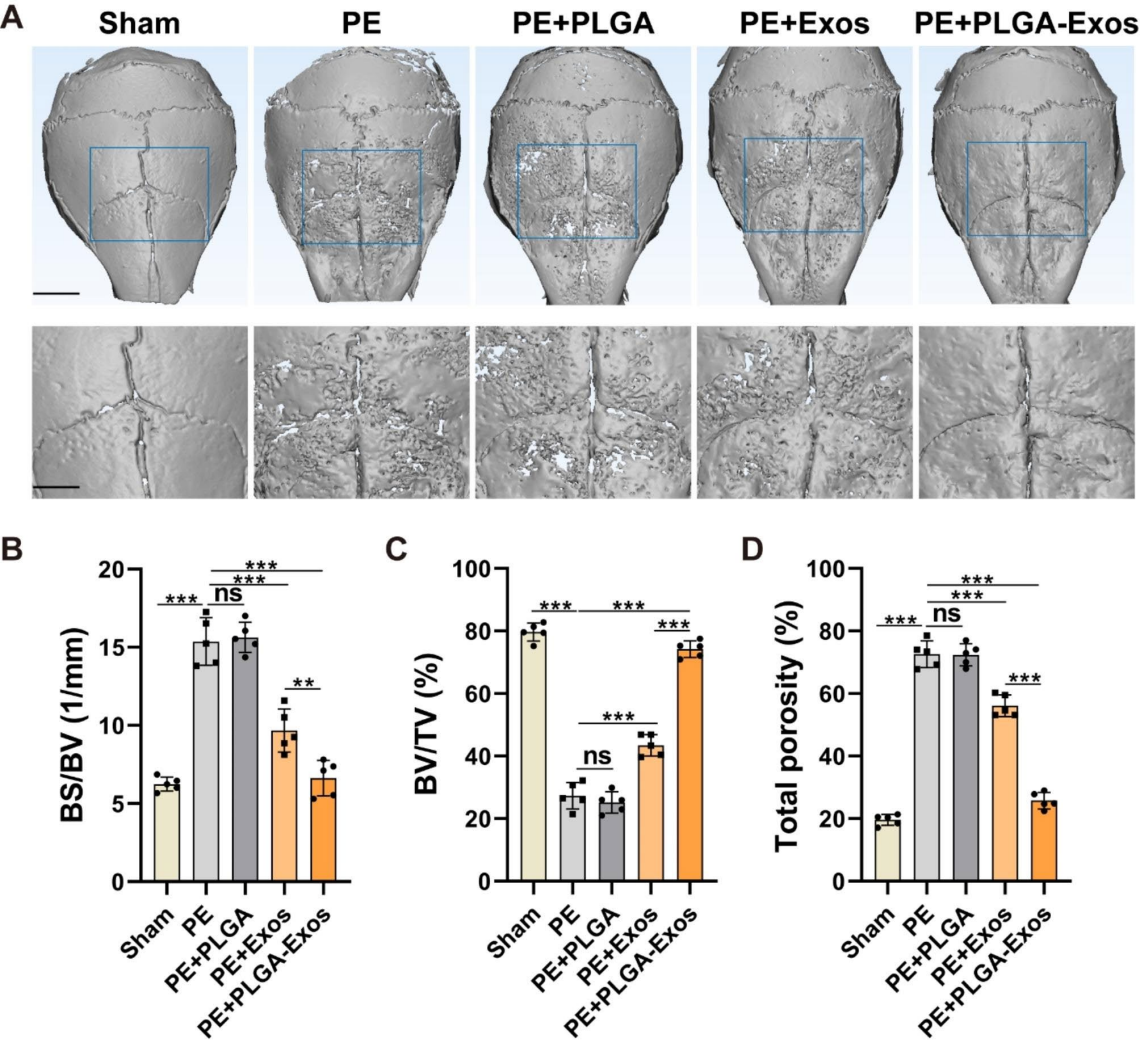
**PLGA-Exos attenuate bone loss in osteolysis mice by micro-CT.** (**A**) Representative micro-CT surface images of the calvarial bone among groups. Scale bar, 5 mm (above) and 2.5 mm (below). Quantitative analyses of the BV/TV (**B**), BS/BV (**C**), and total porosity (**D**). (n = 5 samples per group, ANOVA, *P < 0.05, ** *P* < 0.01, *** *P* < 0.001)

**Fig. 7:**
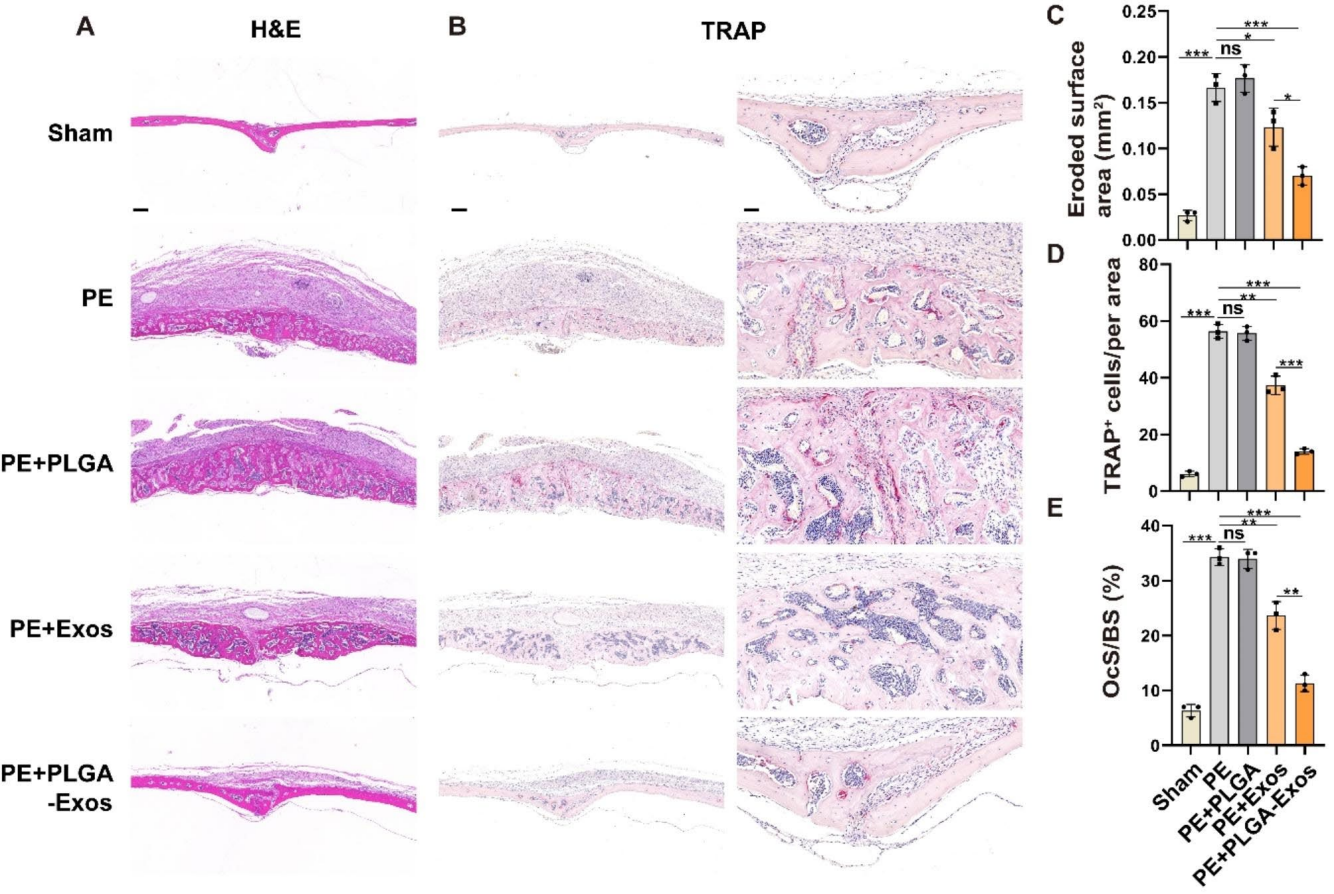
**Histological staining and histomorphometry analysis of calvaria sections.** (**A**) Representative images of H&E staining. Scale bar, 200 μm. (**B**) Representative images of TRAP staining and the corresponding areas were enlarged. Scale bar, 200 µ m (left), 50 µ m (right). (**C**-**E**) Quantitative analyses of eroded surface area, number of TRAP^+^ osteoclasts, and OcS/BS were calculated. (n = 3 samples per group, ANOVA, *P < 0.05, ** *P* < 0.01, *** *P* < 0.001)

**Fig. 8:**
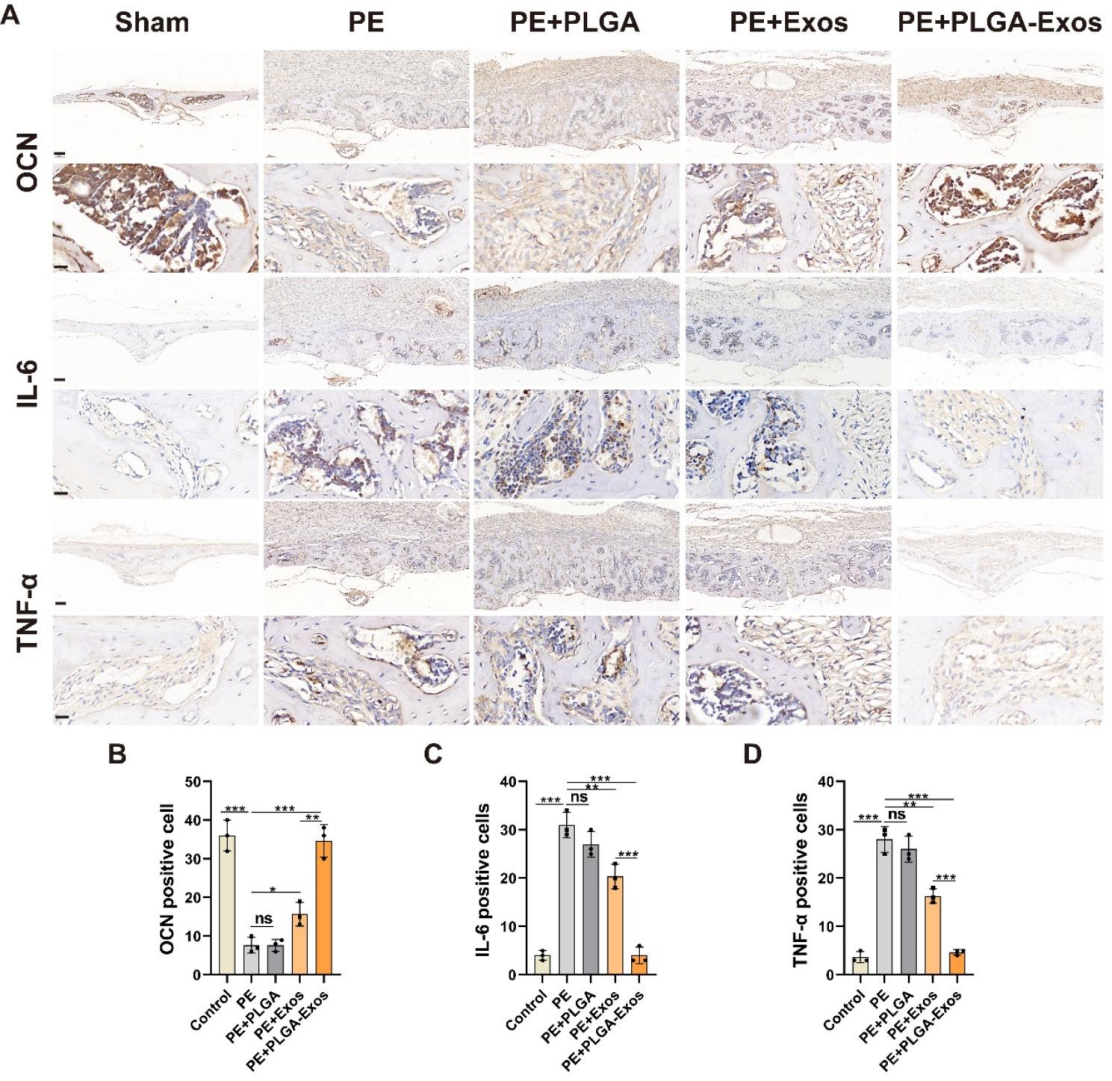
**Immunohistochemical staining and quantitative analysis of OCN, IL-6, and TNF-α in mouse calvaria of each group.** (**A, B**) Representative images of OCN staining and percentage of OCN positive cells (n = 3). Scale bar, 100 μm (above), 16 μm (below) (**A, C**) Representative images of IL-6 staining and percentage of IL-6 positive cells (n = 3). Scale bar, 100 µ m (above), 16 µ m (below) (**A, D**) Representative images of TNF-α staining and percentage of TNF-α positive cells (n = 3). Scale bar, 100 µ m (above), 16 µ m (below). (n = 3 samples per group, ANOVA, *P < 0.05, ** *P* < 0.01, *** *P* < 0.001)

According to a previous report [4], a calvarial osteolysis model was induced in male C57BL/6J mice using PE particles. On the second day after surgery, local injections of PBS, PLGA, Exos, and PLGA-Exos were administered, followed by weekly injections. After one month, the mice were euthanized, and skull slices and major organs were collected. Micro-CT analysis (Fig. 6A) revealed evident bone resorption on the skull surface in the PE group compared to the Sham group, characterized by varying sizes of bone resorption pits. Both the PLGA-Exos group and Exos group significantly mitigated PE particles-induced osteolysis, with the PLGA-Exos group exhibiting a superior inhibitory effect on bone resorption. Further measurements were conducted for BV/TV, bone BS/BV, and total porosity of the skull (Fig. 6B-D). The results demonstrated a significant decrease in BV/TV and an increase in BS/BV and total porosity in the PE group and PE + PLGA group compared to the sham group, indicating substantial osteolysis. In comparison to the Exos group, the PLGA-Exos group exhibited higher BV/TV and lower BS/BV and porosity. H&E staining (Fig. 7A) and optical microscopy were utilized on coronal slices to calculate the eroded surface area, providing histological evidence. PE and PE + PLGA treatments significantly increased the eroded surface area compared to the Sham group, while the PLGA-Exos group showed a better therapeutic effect than the Exos group (Fig. 7C). TRAP staining was employed to evaluate osteoclast formation in skull sections (Fig. 7B), revealing a significant increase in the number of osteoclasts in the PE and PE + PLGA groups. The PLGA-Exos-treated group exhibited fewer osteoclasts and a lower percentage of osteoclasts surface per bone surface (OCs/BS, %) compared to the Exos group (Fig. 7D, E). Immunohistochemistry was performed to detect the expressions of OCN, IL-6, and TNF-α in skull sections (Fig. 8A-D). The results demonstrated low expression of the osteoblast marker OCN and high expression of the inflammatory cytokines IL-6 and TNF-α in the PE and PE + PLGA groups. The PLGA-Exos group exhibited higher expression of OCN and lower expression of IL-6 and TNF-α compared to the Exos group. These findings suggest that the PLGA-Exos group has a superior effect in preventing PE-induced osteolysis in mice compared to the Exos group. H&E staining of the main organs was conducted to evaluate the safety of PLGA-Exos application in the body, revealing no obvious toxic side effect between the PLGA-Exos group and the other groups (Figure.S5).

## Discussion

TJA has emerged as one of the most dependable surgical interventions for individuals suffering from end-stage bone and joint diseases, contributing to a continually rising global trend in the number of joint replacement surgeries performed [29]. However, it is accompanied by the substantial risk of aseptic loosening of the prosthesis, a significant postoperative complication often necessitating intricate revision surgery [30]. The primary culprit behind periprosthetic osteolysis, which leads to aseptic loosening of the prosthesis, is the generation of wear particles resulting from the friction between the implant and the host bone, with PE particles being particularly prevalent [31, 32]. This process triggers the involvement of osteoblasts, osteoclasts, and mesenchymal stem cells, setting the stage for aseptic inflammation around the prosthesis and the subsequent release of a multitude of pro-inflammatory factors [33]. Notably, despite these well-documented processes, there is a conspicuous absence of specific molecular drugs designed for the precise regulation of the osteoclast/osteoblast imbalance within the bone microenvironment surrounding aseptic loosening of the prosthesis. In this context, it is clear that while TJA offers immense promise, the challenge of addressing aseptic loosening of the prosthesis and its complex underlying mechanisms remains a critical area for further research and the development of targeted therapeutic interventions.

The present study has revealed that HucMSCs-Exos exert a superior regulatory effect on bone metabolism. Specifically, HucMSCs-Exos have been shown to play a pivotal role in enhancing fracture healing through the Wnt signaling pathway [34], and HucMSCs-Exos derived miR-365a-5p have demonstrated the ability to promote osteogenesis via the Hippo signaling pathway [35]. Additionally, HucMSCs-Exos have been found to boost the expression of the angiogenic factor vascular endothelial growth factor (VEGF), while exosome-derived miR-21-5p has exhibited the capacity to prevent femoral head necrosis by stimulating angiogenesis and osteogenesis [36, 37]. However, it’s important to note that exosomes have a relatively short in vivo survival time and are susceptible to elimination. Consequently, combining exosomes with biomaterials has become a focal point of research in the development of exosome-based therapeutics. For instance, the combination of HucMSCs-Exos with Pluronic F127 hydrogel has demonstrated the ability to promote wound healing and achieve complete skin regeneration in individuals with chronic diabetes mellitus [38]. Likewise, the incorporation of HucMSCs-Exos into hydroxyapatite-embedded hyaluronic-alginate gel has significantly advanced bone regeneration [39]. In the scope of our study, we successfully prepared PLGA-Exos for the prevention and treatment of periprosthetic osteolysis. Importantly, the PLGA we employed has received approval from both the US Food and Drug Administration (FDA) and the European Medicines Agency (EMA) as a safe substance for clinical use, and it is metabolically excreted by the body in the form of carbon dioxide and water [39]. Our data unequivocally demonstrate that locally released PLGA-Exos can dissolve within bone tissue and maintain their biological activity, offering distinct advantages in the treatment of bone dissolution. Compared to a single local injection of exosomes, PLGA-Exos ensure a more sustained presence in vivo, thereby reducing the frequency of administration required for clinical applications. Moreover, PLGA-Exos exhibits excellent biosafety profiles and can effectively sustain therapeutic exosome concentrations within osteolytic regions. As such, the PLGA-Exos sustained release system holds immense clinical application potential, offering promising prospects for the prevention and treatment of periprosthetic osteolysis.

Our data unequivocally demonstrate the remarkable superiority of PLGA-Exos in treating cranial osteolysis induced by PE particles. Nevertheless, our study is not without its limitations. PLGA nanoparticles can be prepared through various processing methods including double-emulsion solvent evaporation and single-emulsion solvent evaporation. In this study, only single-emulsion solvent evaporation was used to prepare PLGA-Exos and applied to in vivo and in vitro experiments. We need to further analyze the possibility and advantages of double-emulsion solvent evaporation in preparation of PLGA-Exos, and compare the two methods of preparing PLGA-Exos from many aspects, so as to further improve the clinical application transformation of our research results. One notable concern arises from the ultrasonic mechanical shear stress applied during the preparation process, which can potentially induce membrane damage, compromise the integrity of exosome membranes, and significantly reduce their microviscosity [40]. Additionally, this process may lead to the reassembly of exosome vesicles [41]. Addressing these challenges and minimizing their impact on the biological functionality of exosomes requires further investigation. Furthermore, it’s essential to acknowledge that exosomes possess certain immunogenic properties, and different separation methods can influence the purity, quantity, and physicochemical properties of the obtained exosomes [42]. Therefore, it may be prudent to explore the development of individualized therapies based on autologous somatic exosomes while establishing standardized separation and purification procedures, which could prove to be an effective solution. Considering that exosomes are rich sources of functional proteins and miRNAs [43], it is imperative to delve deeper into the precise molecular mechanisms underlying the involvement of PLGA-Exos in osteolysis. This deeper understanding can pave the way for the clinical application of PLGA-Exos in osteolysis. To bring the clinical application of PLGA-Exos for osteolysis to fruition, we must further engineer joint prostheses equipped with PLGA-Exos and rigorously assess their long-term in vivo biosafety. By addressing the challenges, the PLGA-Exos sustained-release system holds the promise of becoming an innovative approach for the clinical utilization of exosomes, offering hope for the effective management of osteolysis in joint replacement procedures.

## Conclusion

In this study, we have successfully engineered and characterized PLGA-Exos to mitigate and treat periprosthetic osteolysis. It is noteworthy that PLGA-Exos effectively retains the inherent biological functions of internal exosomes. Our collected data unequivocally demonstrates that PLGA-Exos have the capacity to continuously release exosomes at the local site of osteolysis, thereby maintaining a therapeutically effective concentration of exosomes precisely where they are needed most. The pivotal role played by PLGA-Exos in preventing and treating periprosthetic osteolysis is twofold. First, they foster the osteogenic differentiation of local BMSCs, promoting bone regeneration. Second, they impede the osteoclastic differentiation of macrophages through the release of exosomes, thereby inhibiting bone resorption. Our research offers a novel approach to clinical interventions for periprosthetic osteolysis, and the PLGA-Exos sustained-release system offers a fresh perspective on the clinical application of therapeutic exosomes.

**Fig. 9:**
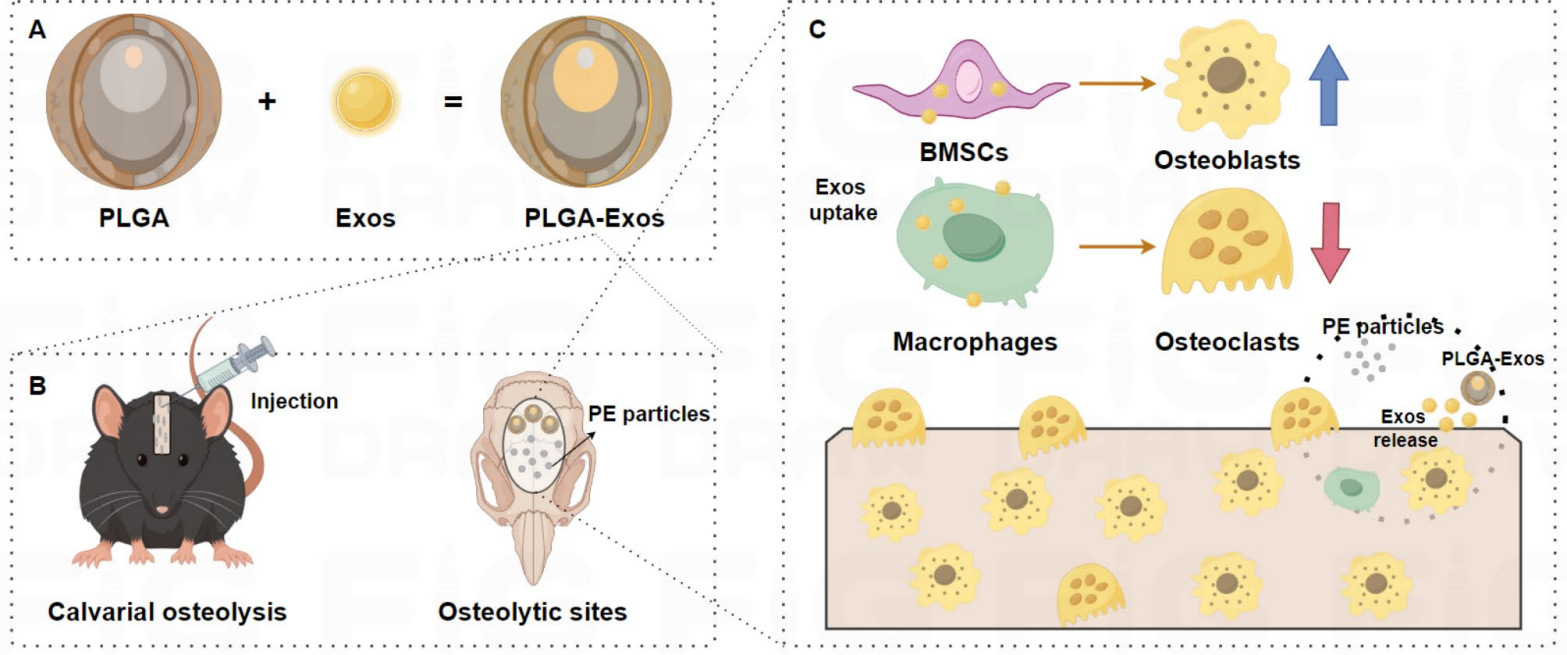
**Schematic diagram of PLGA-Exos alleviates PE particles-induced mouse calvarial osteolysis.** (**A**) Construction of PLGA-Exos. (**B**) Local injection of PLGA-Exos at osteolytic sites. (**C**) PLGA-Exos continuously released exosomes, enhanced osteogenic differentiation, and inhibited osteoclast formation. (By Figdraw)

### Electronic supplementary material

Below is the link to the electronic supplementary material.

Supplementary Material 1

## Data Availability

Not applicable.
