# Supplementary material for: PLGA nanoparticles engineering extracellular vesicles from human umbilical cord mesenchymal stem cells ameliorates polyethylene particles induced periprosthetic osteolysis
Source: J Nanobiotechnology. 2023 Oct 31;21:398. doi: 10.1186/s12951-023-02177-7 (PMC10617042; doi:10.1186/s12951-023-02177-7)
Supplement: Supplementary file 1 — Supplementary Material 1 [file 12951_2023_2177_MOESM1_ESM.docx]

Supplementary Materials for

PLGA nanoparticles engineering extracellular vesicles from human umbilical cord mesenchymal stem cells ameliorates polyethylene particles induced periprosthetic osteolysis


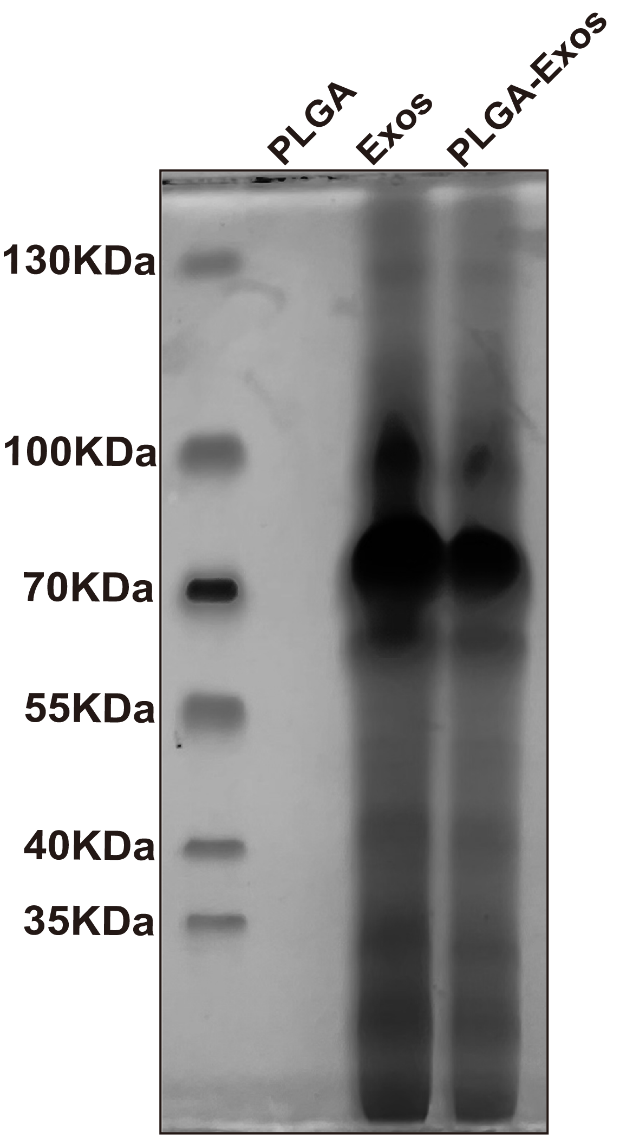


**Figure. S1** Coomassie blue staining of PLGA, Exos, and PLGA-Exos.


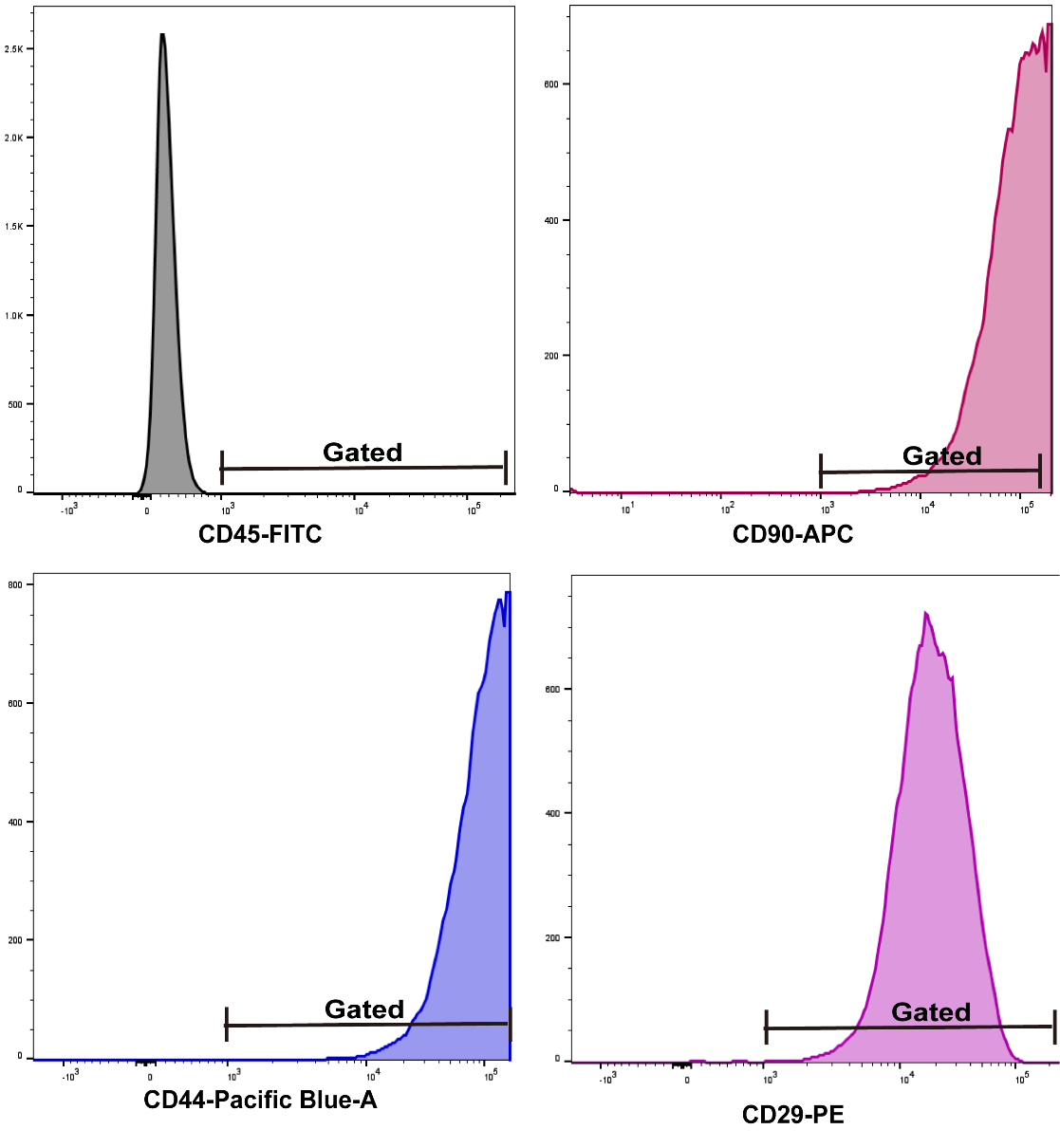


**Figure .S2** Surface markers on BMSCs were determined by flow cytometry analysis.


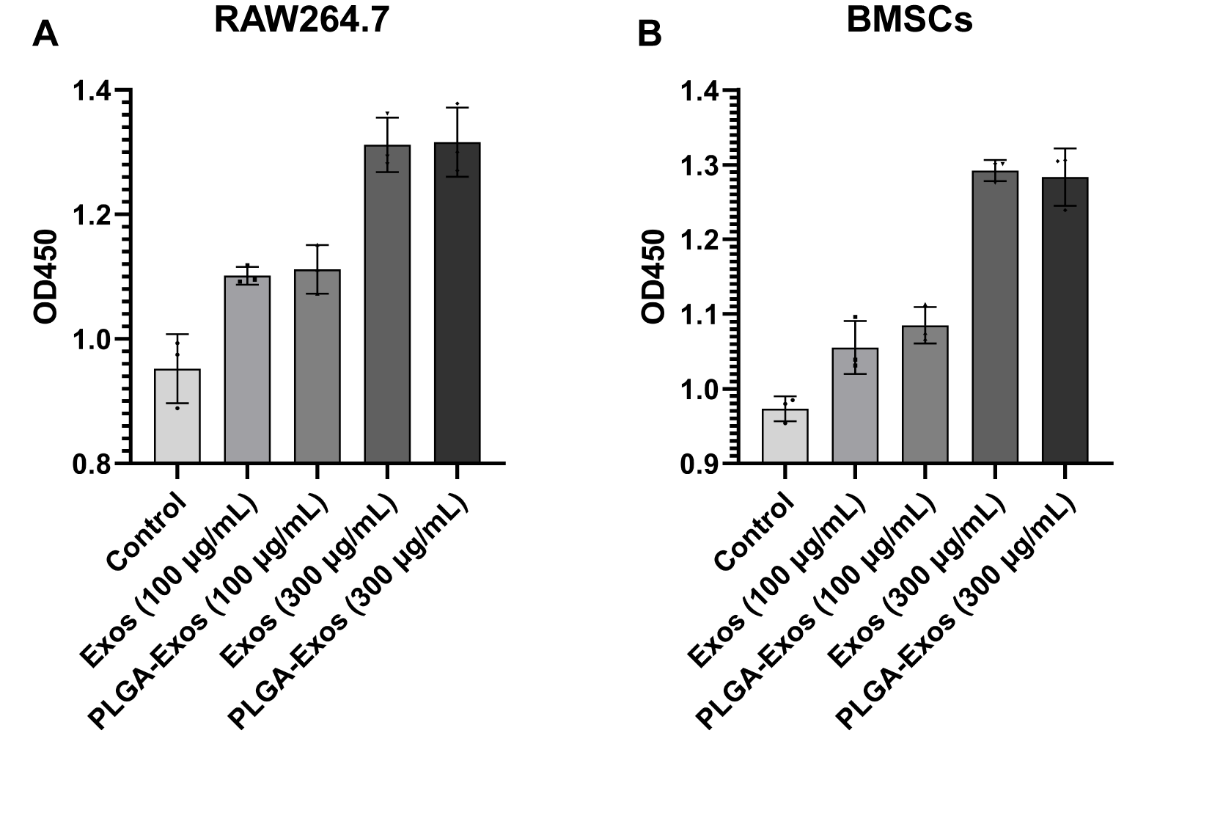


**Figure .S3** The CCK-8 assay was performed of (A) RAW264.7 and (B) BMSCs treated with various concentrations of Exos and PLGA-Exos for 48 h (n =3).


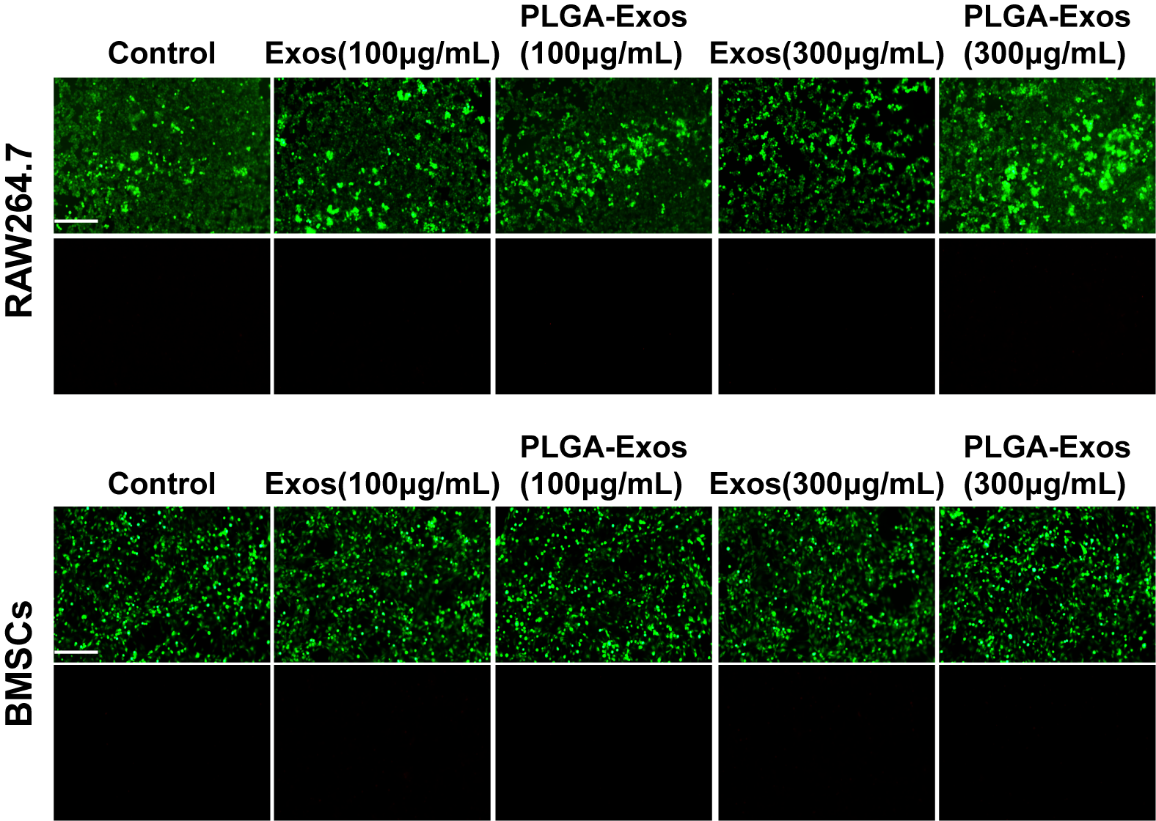


**Figure .S4** The Live/Dead cell double staining of RAW264.7 and BMSCs treated with various concentrations of Exos and PLGA-Exos for 48 h (n = 3). Scale bar: 200 μm.

.


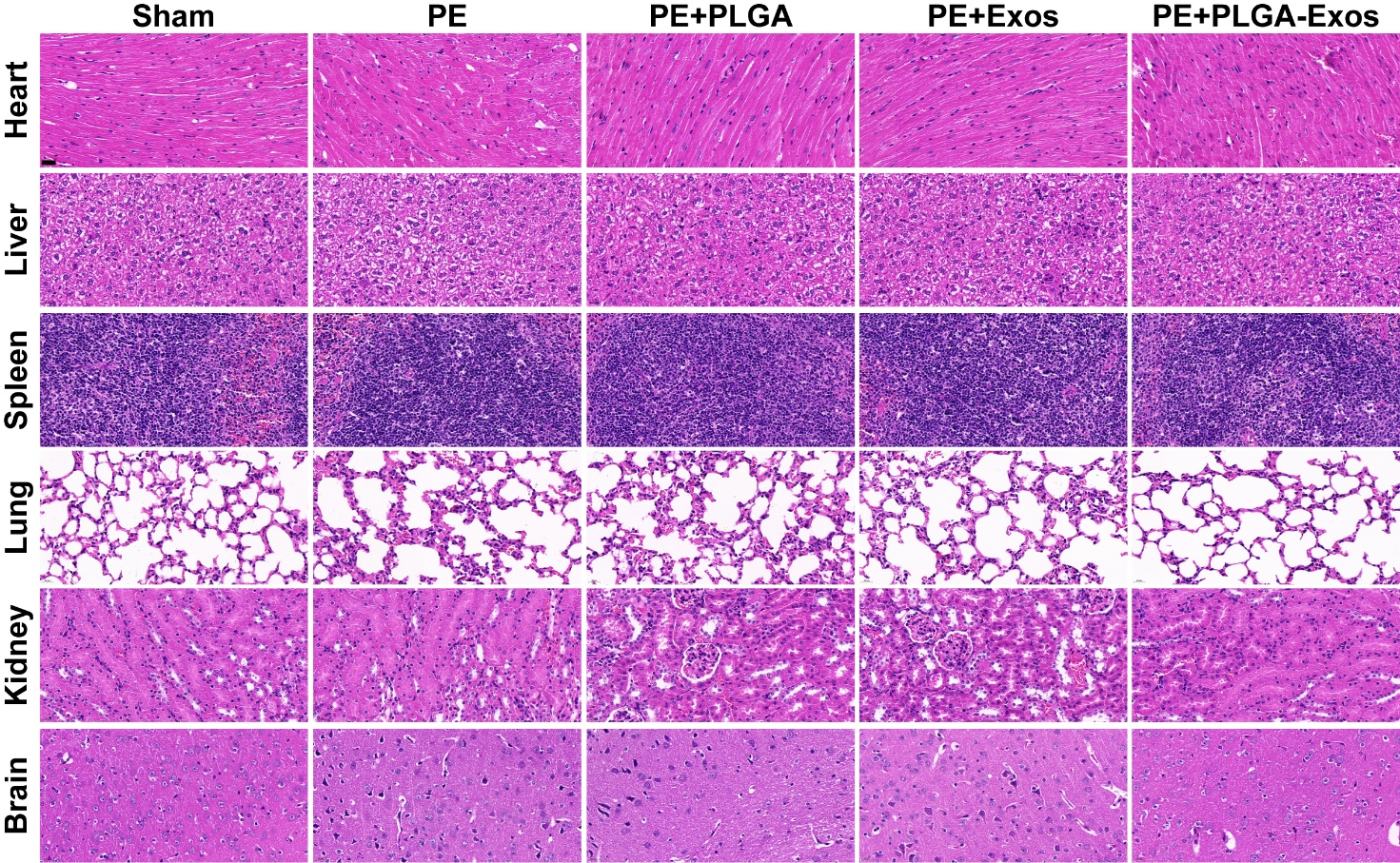


**Figure. S5** H&E staining of major organs after PLGA-Exos treatment. Scale bar, 20µm.
